# Biological and Physicochemical Functions of Ubiquitylation Revealed by Synthetic Chemistry Approaches

**DOI:** 10.3390/ijms18061145

**Published:** 2017-05-27

**Authors:** Daichi Morimoto, Erik Walinda, Kenji Sugase, Masahiro Shirakawa

**Affiliations:** 1 Department of Molecular Engineering, Graduate School of Engineering, Kyoto University, Kyoto-Daigaku Katsura, Nishikyo-ku, Kyoto 615-8510, Japan; morimoto@moleng.kyoto-u.ac.jp (D.M.); sugase@moleng.kyoto-u.ac.jp (K.S.); 2 Department of Molecular and Cellular Physiology, Graduate School of Medicine, Kyoto University, Yoshida Konoe-cho, Sakyo-ku, Kyoto 606-8501, Japan; walinda.erik.6e@kyoto-u.ac.jp

**Keywords:** ubiquitin, post-translational modification, chemical ubiquitylation, site-directed conjugation

## Abstract

Most intracellular proteins are subjected to post-translational modification by ubiquitin. Accordingly, it is of fundamental importance to investigate the biological and physicochemical effects of ubiquitylation on substrate proteins. However, preparation of ubiquitylated proteins by an enzymatic synthesis bears limitations in terms of yield and site-specificity. Recently established chemical ubiquitylation methodologies can overcome these problems and provide a new understanding of ubiquitylation. Herein we describe the recent chemical ubiquitylation procedures with a focus on the effects of ubiquitylation on target proteins revealed by the synthetic approach.

## 1. Introduction

Ubiquitin plays key roles in a myriad of cellular events via covalent conjugation to intracellular proteins. Not only has ATP-dependent protein degradation mediated by ubiquitylation (covalent modification of a protein with ubiquitin) been revealed, but also its non-proteolytic functions in immune response and DNA repair [1]. According to the Protein Information Resource [2] and the PhosphoSite Plus database [3], most eukaryotic intracellular proteins are ubiquitylated at multiple sites. In some cases, polymeric chains of ubiquitin are formed on substrate proteins. These anchored polyubiquitin chains can have various linkage types and lengths. In polyubiquitin chains, either the N-terminal residue (M1) or one of seven internal lysine residues (K6, K11, K27, K29, K33, K48, and K63) of a given ubiquitin molecule is covalently conjugated to the C-terminus of another ubiquitin molecule [1]. Polyubiquitin chains of different linkage types adopt characteristic conformations that are specifically recognized by ubiquitin-binding proteins. By this general mechanism, ubiquitylation can mediate a wide variety of cellular functions; the position of the ubiquitylation site on a given substrate protein and the linkage type of the polyubiquitin chains are the critical determinants for the signal transduction process.

To study the function of ubiquitylation and its effect on target proteins, it is necessary to prepare sufficient amounts of ubiquitylated proteins. Initially, the fusion expression method had been established to mimic ubiquitylation [4]. In this approach, an uncleavable ubiquitin moiety (ubiquitin G76V) is fused to the N-terminus of a target protein, which induces the ubiquitin fusion degradation (UFD) pathway. The fusion protein is specifically degraded by the proteasome via the recognition of the conjugated ubiquitin moiety by downstream receptor proteins. In fact, this kind of ubiquitylation via the N-terminus has been reported to occur in vivo in some proteins, such as the myoblast determination protein (MyoD) and the cell-cycle regulator p21 [5], whereas in most cases an isopeptide bond between a lysine residue on the substrate protein and the C-terminal glycine residue of ubiquitin is formed. In living cells, ubiquitin conjugation occurs by successive enzymatic reactions involving ubiquitin-activating (E1), ubiquitin-conjugating (E2), and ubiquitin ligase (E3) enzymes [6,7]. Therefore, in cases where all necessary enzymes are identified and available in sufficient amounts, the ubiquitylation reaction can be reconstituted in vitro. In addition, it is also possible to obtain ubiquitylated protein samples using co-expression of ubiquitin, substrate proteins and the E1, E2, and E3 enzymes in *Escherichia coli* [8]. However, these enzymatic approaches are not applicable to all cases and it is difficult to regulate polyubiquitylation activity. For instance, even though mono-ubiquitylated proteins are required for a given study, polyubiquitylated proteins would also be obtained; therefore, additional purification steps would be required.

On the other hand, a number of chemical ubiquitylation methods have been reported in the last decade. Compared to enzymatic methods, the chemical approaches are more adjustable with respect to the site of ubiquitylation and the length of polyubiquitin chains. In particular, the chemical approaches present powerful strategies in cases where the E2 and E3 enzymes are not identified or are difficult to prepare. In this review, we discuss recently-established methods of chemical ubiquitylation focusing on the effects of ubiquitylation on target proteins achieved by the synthetic approach.

## 2. Strategies for Chemical Preparation of Ubiquitylated Proteins

Many kinds of synthetic methods have been proposed not only for ubiquitylated proteins, but also for polyubiquitin chains [9]. We summarize the recently-established biological and chemical ubiquitylation strategies, in which the chemical forms of donor (distal) ubiquitin and acceptor molecules are described, along with the character of the linkage as compared with native ubiquitylation (Table 1). Here, we will briefly review the main concepts; more in-depth discussions on chemical ubiquitylation methods have been presented elsewhere [10,11,12,13,14,15,16,17].

### 2.1. Native Chemical Ligation and Isopeptide Chemical Ligation

In the preparation of ubiquitylated proteins, the main challenge was to establish a strategy for specific isopeptide bond formation between the lysine residue of a given protein and the C-terminal glycine residue of ubiquitin. One of the most suitable chemical reactions for the isopeptide bond formation is a chemoselective reaction termed native chemical ligation (NCL). In 1994, Dawson and co-workers introduced NCL, in which a selective peptide bond is formed between a given peptide containing a C-terminal α-thioester group and another peptide containing a cysteine residue [60]. The specificity of NCL makes it a proper method for ubiquitylation by chemical synthesis. In fact, Chatterjee and co-workers utilized NCL for chemical ubiquitylation in 2007, which was the first report of a chemical ubiquitylation method [43]. In this method, a recombinantly-expressed ubiquitin-α-thioester was ligated to the side chain of a lysine residue of the histone H2B peptide with an auxiliary group. The photolabile auxiliary group was removed by ultraviolet (UV) irradiation and a traceless native isopeptide bond between the lysine residue of histone H2B and the C-terminal glycine residue of ubiquitin was formed. This auxiliary-mediated NCL method requires the auxiliary group to be conjugated on the lysine residue (achieved by using solid-phase peptide synthesis (SPPS)). Moreover, protein denaturation by guanidine hydrochloride is also necessary for the ligation between the peptide and ubiquitin. Therefore, the applicability of this method is limited to intrinsically-disordered proteins, such as N-terminal fragments of histones and α-synuclein, or the proteins that are easily refolded from the denatured state.

Li and co-workers introduced a genetic incorporation of a pyrrolysine analogue into a target protein by using the pyrrolysine tRNA and its synthetase [40]. The advantage of this methodology is that it does not require protein denaturation for the ligation. However, in this method, the product contains non-native ubiquitin with a cysteine at residue 76 (ubiquitin G76C). In 2010, Virdee and co-workers solved this problem by genetic incorporation of the unnatural amino acid *N*ε*-*(*t-*butyloxycarbonyl)-l-lysine [56]. In this methodology, termed “genetically encoded orthogonal protection and activated ligation” (GOPAL), the isopeptide bond is formed through Ag^+^-catalyzed condensation. Importantly, this method does not involve any steps of protein denaturation and allows a traceless isopeptide bond to be formed between a target protein and ubiquitin [55].

### 2.2. Ubiquitin Conjugation via Non-Native Linkages

**Figure 1:**
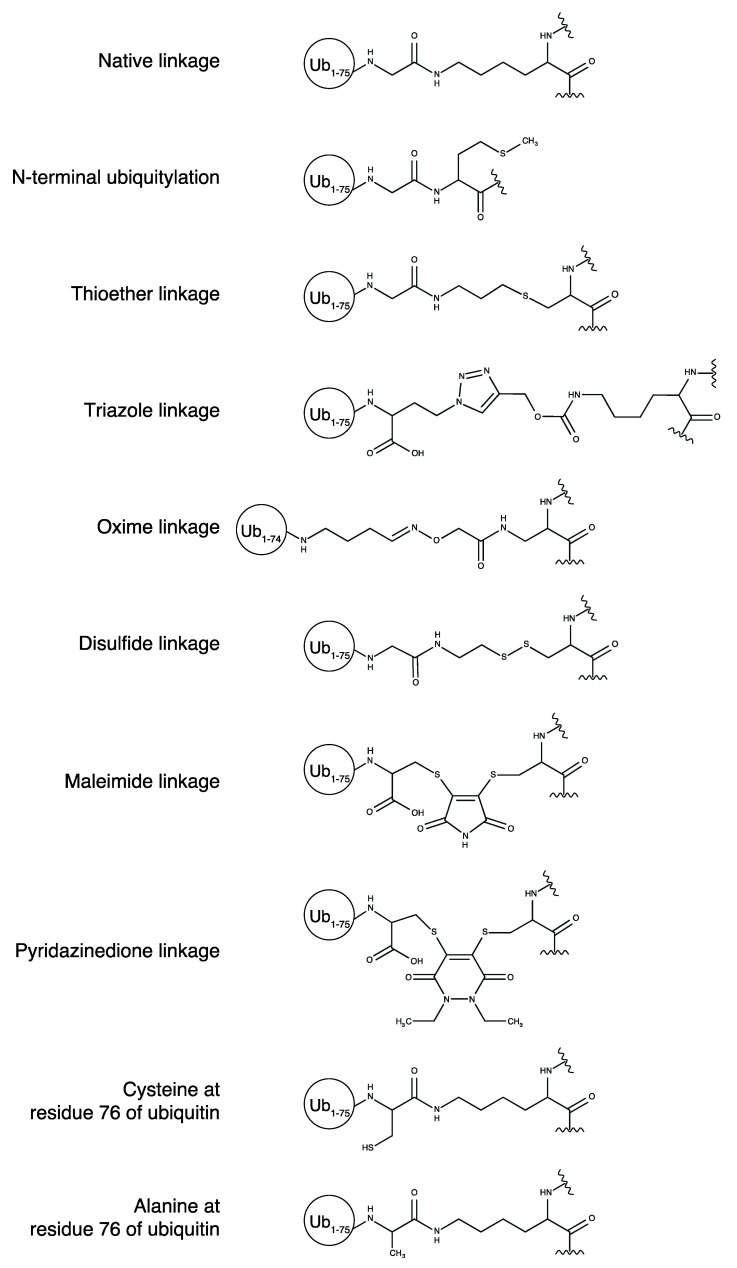
Differences in linkage structure between native and chemical ubiquitylation. Since there is variation in the reported chemical ubiquitylation methods using thioether, triazole, and disulfide linkages, representative structures are shown as follows: thioether linkage: Valkevich et al. 2012 [18]; triazole linkage is Eger et al. 2011 [22]; disulfide linkage: Chen et al. 2010 [28].

Scientists also developed other synthetic approaches for the chemical ubiquitylation in which an isopeptide bond between a target protein and ubiquitin is not utilized. Thioester [18,19,20], triazole [21,22,23,24], oxime [25], disulfide [26,27,28,29,30,31,32,33], maleimide [34], and pyridazinedione linkages [34] are used instead of isopeptide bonds. The methods using these non-native linkages are relatively straightforward and appropriate for the preparation of large amounts of ubiquitylated proteins. In addition, the non-native isopeptide linkages are not cleaved by deubiquitinating enzymes (DUBs), which allows the determination of the binding affinity of DUBs for the synthesized polyubiquitin chains [25]. However, the limitations of these non-native linkages are that their chemical properties (linkage flexibility, length, or bulkiness) are different from those of the native isopeptide linkage (Figure 1). When investigating direct physicochemical effects of ubiquitylation on target proteins, it is highly important to consider such differences in chemical composition.

One of the most popular non-native linkage conjugations is disulfide conjugation. In this method, it is necessary to mutate the lysine residue to be ubiquitylated to a cysteine residue in a target protein. In addition, one must replace its solvent-exposed intrinsic cysteine residues by mutation to other amino acids. The disulfide bridge is formed between the C-terminal thiol group of ubiquitin G76C and the newly-introduced thiol group on the target protein. Importantly, this methodology does not involve any protein denaturation steps and is a simple, straightforward, and versatile approach. In fact, the disulfide-mediated ubiquitylation has been applied to many kinds of folded proteins (Table 1).

## 3. Biological Effects of Ubiquitylation on Substrates

### 3.1. Recognition of Ubiquitin Tags

**Figure 2:**
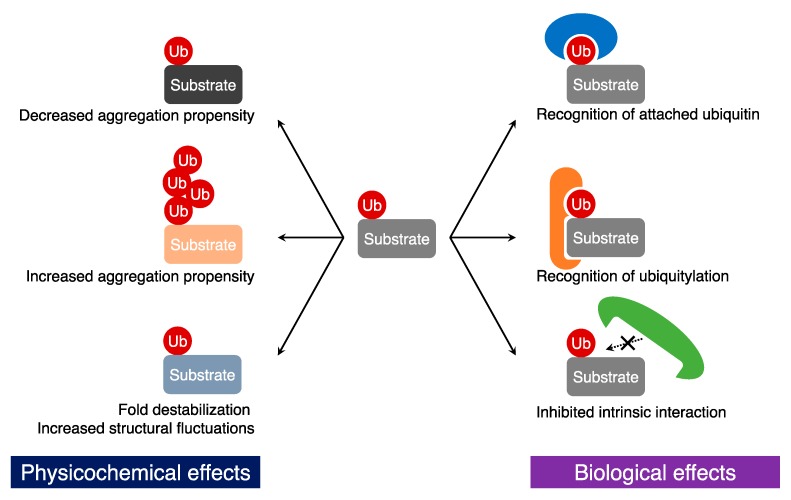
Biological and physicochemical effects of ubiquitylation on conjugated proteins. The right blue, orange, and green graphics: ubiquitin-binding proteins; Ub: ubiquitin.

The attached ubiquitin moieties on a substrate protein can be recognized by ubiquitin-binding proteins [61] (Figure 2). Similar to the UFD pathway, chemical ubiquitylation also functions as a degradation signal via recognition of the attached ubiquitin molecules. Indeed, ubiquitylation-induced proteasomal degradation of cyclin B [21], α-globin [33], and α-synuclein [39] has been observed. In general, (poly-)ubiquitin tagged proteins are recruited to the 26S proteasome via recognition of the ubiquitin tags and degraded in the proteasomal core. In many cases, K48-linked polyubiquitylation mediates ATP-dependent proteasomal protein degradation [62]. The proteasomal degradation of α-globin in vitro has clearly indicated that mono-ubiquitylation is not sufficient as a proteolytic signal and that K48-linked polyubiquitylation induces the proteasomal degradation [33]. On the other hand, in some cases, other linkage-type polyubiquitin chains appear to regulate protein degradation. Schneider and co-workers revealed that the addition of chemically-synthesized K11-linked polyubiquitin chains into extracts of *Xenopus* eggs prevented degradation of cyclin B. In addition, they indicated that the treatment with the K11-linked polyubiquitin chains perpetuated the meiotic state, which is consistent with the fact that K11-linked polyubiquitylation is involved in cell cycle regulation via protein degradation [63].

A chemical ubiquitylation study by Shabek and co-workers revealed that mono-ubiquitylation induces proteasomal degradation of a poly-peptide of ~150 residues without additional ubiquitylation [39]. As discussed above, in many cases, polyubiquitylation seems to be necessary for proteasomal degradation. However, mono-ubiquitylation is sufficient for the proteasomal degradation in the cases of moderately sized proteins (~150 residues); in addition, the conjugated peptide is also required to be longer than 20 residues [39,64]. A single ubiquitin moiety is sufficient to target such moderately-sized substrate proteins to the proteasome; on the other hand, in the cases of larger proteins, longer polyubiquitin chains may be necessary for proteasomal targeting. Therefore, in the ubiquitin-proteasome pathway, long polyubiquitin chains are not always necessary to initiate degradation. The size of the substrate proteins may be one of the determinants for ubiquitylation-mediated protein degradation. Although it might be difficult to evaluate such mono-ubiquitylation-driven proteasomal degradation by using enzymatic approaches, the chemical synthetic method clearly revealed the recognition of the attached mono-ubiquitin for degradation and the size-regulation in the proteasomal degradation system.

### 3.2. Generation of New Protein-Protein Interactions

Some of the ubiquitin attachments to target proteins lead to new protein-protein interactions that are unrelated with protein degradation (Figure 2). The chemical mono-ubiquitylation of proliferating cell nuclear antigen (PCNA) enhances the interaction with DNA polymerase η (polη), causing a decrease in DNA synthesis [22,28]. Although the biological role of this mono-ubiquitylation of PCNA had been well studied before [65,66], the chemical ubiquitylation allowed to investigate mono-ubiquitylation of PCNA at various positions [28]. The conformation of the attached ubiquitin moiety appeared to be flexible and, therefore, the mono-ubiquitylation of PCNA at any site observed appeared to decrease the rate of DNA synthesis. The results suggested that the conformational flexibility of ubiquitin enables the mediation of a long-range protein-protein interaction among ubiquitin, PCNA, and polη.

Similar to mono-ubiquitylation of PCNA, histone H2B increased the interaction with the H3K79 methyltransferase hDot1L [26,44,45] and mouse nucleosome assembly protein 1 (mNap1) [42]. The chemical ubiquitylation method allowed sample preparation in high amounts, resulting in reconstitution of a nucleosome containing ubiquitylated histones. Intriguingly, the stability analysis of the ubiquitylated nucleosome indicated that ubiquitylation of the H2A–H2B dimer destabilized the nucleosome due to the increased affinity for mNap1 [42]. The interaction of ubiquitylated H2A–H2B with mNap1 may disturb the interactions necessary for nucleosome formation: H2A–H2B:(H3–H4)_2_ or H2A–H2B:DNA interactions.

### 3.3. Inhibition of Protein-Protein Interactions

In contrast to ubiquitylation-induced protein-protein interactions, ubiquitylation may inhibit native protein-protein interactions of the conjugated protein (Figure 2). In the cases of calmodulin [40], human Dishevelled segment polarity protein 2 (Dvl2) Dishevelled-Axin (DIX) domain [41], and Ras [31], ubiquitin obstructs the interface on the modified proteins with other proteins. In particular, Madrzak and co-workers observed that mono-ubiquitylation attenuates the formation of filament assembly of the DIX domain [41]. The DIX domain has a tendency of head-to-tail homo(hetero)-polymerization [67]. However, the crystal structure of the DIX domain indicated that the two ubiquitylation sites (K54 and K58) are located within the DIX tail region, suggesting that ubiquitylation blocked the DIX–DIX interface. Indeed, the authors experimentally observed that the DIX filament assembly was severely inhibited by ubiquitylation.

In addition to such a “blocking” effect on a protein due to attached ubiquitin molecules, ubiquitylation could create steric clashes in a protein complex. In formation of a nucleosome, ubiquitylation may indeed exert structural influences on histone H2B [58]. The cryo-electron microscopy structure of the reconstituted nucleosome containing ubiquitylated H2B indicated that the two flexible ubiquitin moieties protruded in between nucleosome-wrapping DNA chains. In addition, a thermal stability assay showed that the peak corresponding to the H2A-H2B form of the nucleosome was shifted toward a slightly lower temperature, suggesting that the nucleosome containing ubiquitylated H2B was destabilized. Thus, the attached ubiquitin moieties may disturb the structural properties of the conjugated protein and thereby affect protein-protein interactions.

## 4. Physicochemical Effects of Ubiquitylation on Target Proteins

### 4.1. Changes in Aggregation Propensity

Studies that employ chemical ubiquitylation have examined not only biological effects, but also physicochemical effects of ubiquitylation. Ubiquitin has an extraordinary structural rigidity and high solubility in vitro. Therefore, it appears that ubiquitylation has the potential to affect the physicochemical properties of conjugated proteins (Figure 2). Indeed, it has been shown that mono-ubiquitylation inhibits fibril formation of α-synuclein [29,37], but promotes its oligomerization [29]. Meier and co-workers investigated the oligomerization and fibril formation properties of α-synuclein chemically mono-ubiquitylated at various positions using dot blotting and a Thioflavin-T fluorescence assay, respectively [29]. Fibril formation was inhibited in many of the mono-ubiquitin modifications (ubiquitylation at K6, K12, K21, K32, K34, K43, and K96); however, some ubiquitylation sites (ubiquitylation at K10 and K23) resulted in fibril formation levels comparable to wild-type α-synuclein, thereby highlighting the importance of the site of ubiquitylation in affecting aggregation. Interestingly, ubiquitylation of α-synuclein at K96 promoted oligomerization, but not fibril formation. Thus, ubiquitylation of α-synuclein has a site-specific profile in terms of oligomerization and aggregate formation. Importantly, these site-specific mechanistic insights were greatly facilitated by the chemical approach to ubiquitylation that allowed the generation of protein samples ubiquitylated at different sites in large quantities.

Unlike mono-ubiquitylation, polyubiquitylation of α-synuclein was reported to enhance aggregate formation [38] (Figure 2). Lewy bodies in Parkinson’s disease patients are well known to contain insoluble aggregates of polyubiquitylated α-synuclein [68,69]. To understand the role of polyubiquitin in Lewy body formation, Haj-Yahya and co-workers investigated the aggregation profile of tetra-ubiquitylated α-synuclein using chemical ubiquitylation [38]. After incubation at 37 °C with shaking, unmodified α-synuclein remained soluble for 48 h, whereas K48-linked tetra-ubiquitylated α-synuclein rapidly formed soluble, but sodium dodecyl sulfate (SDS)-resistant, aggregates. In addition, wild-type α-synuclein formed mature amyloid fibrils by shaking; in stark contrast, tetra-ubiquitylated α-synuclein formed amorphous aggregates that did not have the morphology of amyloid fibrils. Thus, ubiquitylation largely affects the aggregation propensity of α-synuclein. Intriguingly, this effect strongly depends on the length of the ubiquitin chain and the site of ubiquitylation.

### 4.2. Fold Destabilization and Structural Fluctuation

The molecular weight of ubiquitin (8.6 kDa) is considerably higher than that of other typical post-translational modifiers, such as acetyl (43 Da), methyl (15 Da), and phosphate (97 Da) groups. Therefore, in addition to the ubiquitylation-induced effects on the solubility and aggregation propensity discussed above, ubiquitylation may also exert another influence on the physical properties of target proteins. In fact, a recent molecular dynamics study by Hagai and co-workers indicated that ubiquitylation may induce partial unfolding of target proteins [70]. For this reason, our group has experimentally investigated whether ubiquitylation affects thermodynamic stability and structural dynamics of the modified proteins [32] (Figure 2). After employing disulfide-mediated ubiquitylation to prepare ubiquitylated proteins, tryptophan fluorescence analysis indicated that the thermal transition temperatures of calmodulin, fatty acid binding protein 4 (FABP4), and FK506-binding protein (FKBP12) were decreased by mono-ubiquitylation. Intriguingly, the ubiquitylation-induced fold destabilization depended on the site at which the ubiquitin moiety was attached. Ubiquitylation of a residue located in a β-sheet resulted in larger fold destabilization than ubiquitylation of a loop residue; by contrast, ubiquitylation of a residue located in an α-helix had no significant effect on fold stability. Thus, covalent conjugation of a ubiquitin moiety to the target proteins decreased the fold stability of the target protein and the degree of this destabilization depended on the site of ubiquitylation.

Furthermore, our group detected changes in the structural dynamics of proteins due to ubiquitylation. Based on the chemical shift analysis of peaks in the ^1^H–^15^N hetero-nuclear single quantum coherence spectra, the average tertiary structures of the ubiquitylated proteins were not significantly affected. On the other hand, derivation of the spectral density functions in conjunction with ^15^N relaxation dispersion experiments indicated that ubiquitylation exerted an influence on the motion of the conjugated proteins on the millisecond timescale [32]. These data suggest that the increase in structural fluctuations may explain the lower fold stability of the ubiquitylated protein.

## 5. Conclusions

Today, it is well-known that ubiquitylation regulates the lifetime, enzymatic activity, and localization of proteins. Almost all intracellular proteins are subjected to ubiquitylation and a high number of specific E3 ligases are thought to mediate ubiquitylation. Mass spectroscopic studies have identified more and more ubiquitylation sites of proteins. However, the connection of E3 ligases to their target substrate proteins is not yet sufficiently established. Therefore, chemical ubiquitylation is a powerful and invaluable tool for the preparation of large amounts of ubiquitylated protein samples. As discussed above, diverse chemical approaches have been proposed, which will help to establish a comprehensive understanding of the biological and physicochemical properties of ubiquitylation. In addition, new aspects of ubiquitylation are being continuously uncovered. One of them is the finding of branched polyubiquitin chains [71,72,73]. Ubiquitylation appears to be a much more complicated signal system than expected, compared to when ubiquitin-mediated ATP-dependent proteolysis was first discovered approximately 40 years ago [74]. The established protocols for chemical ubiquitylation will accelerate biological and physicochemical studies of ubiquitylation and we anticipate further development of even more versatile methods of chemical ubiquitylation.

## Figures and Tables

**Table 1:** Established methods to prepare samples of ubiquitylated proteins.

Method	Donor Ub	Acceptor	Difference	Reference
Co-expression	Ub ^wt^	Mind bomb	–	Keren-Kaplan et al. 2012 [8]
Fusion proteins	Ub ^G76V^	β-gal	N-terminal ubiquitylation	Johnson et al. 1995 [4]
Alkene-thiol reaction/chloroketone-thiol	Ub alkene	Ub ^Cys^	Thioether linkage	Valkevich et al. 2012 [18]
	Ub ^Cys^ allylamine	Ub ^Cys^ allylamine		Trang et al. 2012 [19]
	Ub ^G76C^	Ub ^Cys^		Yin et al. 2000 [20]
Alkyne-azide reaction	Ub azide	Polyβ ^Plk^	Triazole linkage	Schneider et al. 2014 [21]
		PCNA ^Plk^		Eger et al. 2011 [22]
		Ub ^Plk^		Eger et al. 2010 [23]
	Ub alkyne	Ub ^AzF^		Weikart et al. 2012 [24]
Aminoxy-aldehyde reaction	Ub aldehyde	Ub ^aminoxy^	Oxime linkage	Shanmugham et al. 2010 [25]
Disulfide conjugation	Ub ^C-terminal amin^^o^^ethanethi^^o^^l linker^	Histone H2B ^Cys^	Disulfide bridge	Chatterjee et al. 2010 [26]; Fierz et al. 2011[27]
		PCNA ^Cys^		Chen et al. 2010 [28]
		α-synuclein ^Cys^		Meier et al. 2012 [29]; Abeywardana et al. 2013 [30]
	Ub ^G76C^	Ras ^Cys^		Baker et al. 2013 [31]
		CaM ^Cys^, FABP4 ^Cys^, FKBP12 ^Cys^		Morimoto et al. 2016 [32]
	Ubhydrazide	α-globin ^Cys^		Hemantha et al. 2014 [33]
Maleimide-thiol reaction	Ub ^G76C^	Ub ^Cys^	Maleimide linkage Pyridazinedione linkage	Morgan et al. 2015 [34]
NCL/ICL	Ub-α-thioester	α-synuclein ^δ^^-thi^^o^^lysine^	–	Kumar et al. 2009 [35]; Erlich et al. 2010 [36]; Hejjaoui et al. 2011 [37]; Haj-Yahya et al. 2013 [38]; Shabek et al. 2012 [39]
		CaM ^pyrr^^o^^lysine anal^^o^^gue^	Cysteine at residue 76 of ubiquitin	Li et al. 2009 [40]
		Dvl2 DIX *^N^*^ε*−*(*t−*butyl^^o^^xycarb^^o^^nyl)−l−lysine^	–	Madrzak et al. 2015 [41]
		Histone H2A *^N^*^ε−(D−cysteinyl)−l−lysine^	Alanine at residue 76 of ubiquitin	Fierz et al. 2012 [42]
		Histone H2B ^thi^^o^^l−auxiliary^	–	Chatterjee et al. 2007[43]; McGinty et al. 2008[44]
			Alanine at residue 76 of ubiquitin	McGinty et al. 2009 [45]
		Peptide ^γ^^−thi^^o^^lysine^	–	Yang et al. 2009 [46]
		Peptide ^thi^^o^^l−auxiliary^		Weller et al. 2014 [47]
		Ub ^azid^^o^^n^^o^^rleucine^		Yang et al. 2014 [48]
		Ub ^B^^o^^c−lysine^		Castaneda et al. 2011 [49]
		Ub ^γ^^−thi^^o^^lysine^		Yang et al. 2010 [50]
		Ub ^δ/γ^^−thi^^o^^lysine^		Merkx et al. 2013 [51]
		Ub ^δ^^−thi^^o^^lysine^		Kumar et al. 2010 [52]; Kumar et al. 2011 [53]; Bavikar et al. 2012 [54]; Virdee et al. 2011 [55]
		Ub *^N^*^ε*−*(*t−*butyl^^o^^xycarb^^o^^nyl)−l−lysine^		Virdee et al. 2010 [56]
	Ub Cys-Pro-ester	Histone H3 ^Cys^	Thioether linkage	Kawakami et al. 2017 [57]
	Ub hydrazide	Histone H2B ^thi^^o^^l−auxiliary^	–	Li et al. 2017 [58]
		Ub ^glycyl−auxiliary^		Pan et al. 2016 [59]

AzF: *p*-azidophenylalanine; Cys: cysteine mutants; CaM: calmodulin; DIX: Dishevelled-Axin; Dvl2: Dishevelled segment polarity protein 2; FABP4: fatty acid binding protein 4; FKBP12: FK506-binding protein; G76C: glycine-to-cysteine mutation at residue 76; ICL: isopeptide chemical ligation; Mdm2: murine double minute 2; NCL: native chemical ligation; PCNA: proliferating cell nuclear antigen; Plk: pyrrolysine analogue; SUMO: small ubiquitin-related modifier; Ub: ubiquitin; wt: wild type. Each linkage structure of ubiquitylation is shown in Figure 1.

## Abbreviations

DUBs	Deubiquitinating enzymes
E1	Ubiquitin-activating enzymes
E2	Ubiquitin-conjugating enzymes
E3	Ubiquitin ligases
NCL	Native chemical ligation
UFD	Ubiquitin fusion degradation
